# Low Adherence to the Mediterranean Diet in Isolated Adolescents: The Mediation Effects of Stress

**DOI:** 10.3390/nu10121894

**Published:** 2018-12-03

**Authors:** Rosario Ferrer-Cascales, Natalia Albaladejo-Blázquez, Nicolás Ruiz-Robledillo, María Rubio-Aparicio, Ana Laguna-Pérez, Ana Zaragoza-Martí

**Affiliations:** 1 Department of Health Psychology, Faculty of Health Science, University of Alicante, 03690 Alicante, Spain; rosario.ferrer@ua.es (R.F.-C.); nicolas.ruiz@ua.es (N.R.-R.); maria.rubio@ua.es (M.R.-A.); 2 Department of Nursing, Faculty of Health Science, University of Alicante, 03690 Alicante, Spain; ana.laguna@ua.es (A.L.-P.); ana.zaragoza@ua.es (A.Z.-M.)

**Keywords:** Mediterranean Diet, loneliness, stress, adolescence

## Abstract

Loneliness perception during adolescence has been increased dramatically in recent years. Changes in lifestyle and difficulties in social interaction could explain this increased phenomenon. As described in previous research, this fact has been associated with the development of high stress levels and dysfunctional lifestyles, in which eating habits play a main role. In this regard, loneliness has been classically associated with poor eating habits, fundamentally the consumption of processed food with little nutritional value. However, the relationship between loneliness and healthy eating patterns, such as the Mediterranean Diet (MD), has not been previously analyzed. The main aim of the present study was to identify the relationship between perceived loneliness, stress, dietary habits, and adherence to the MD in a sample of 527 Spanish adolescents. The obtained results show a significant association between high perceived loneliness and high stress levels with lower MD adherence. Hence, adolescents with high perceived loneliness exhibit poor dietary habits in comparison to those counterparts with low perceived loneliness. Mediation analyses demonstrated an indirect effect of the loneliness on adherence to the MD through the mediation effect of stress. These findings point out a possible mechanism that underlies the classic association between loneliness and health deterioration, based on a poor adherence to a healthy dietary pattern, such as the MD.

## 1. Introduction

The Mediterranean Diet (MD), a recognized healthy dietary pattern, is characterized by being rich in plant food (fruits, cereals, vegetables, legumes, nuts), with a significant intake of olive oil, a moderate consumption of fish, seafood, eggs, poultry, and dairy products, along with a low consumption of red meats [1,2,3]. In recent years, the MD, usually consumed by Mediterranean populations, has been considered as one of the healthiest eating patterns in the world [4]. Several studies have shown the beneficial effects of this dietary pattern on the psychological and physical health across the lifespan. In children and adolescents, high adherence to the MD has been related to better health-related quality of life, academic performance and sleep quality, among others [5,6,7]. During adolescence and adulthood, adherence to the MD has been found to be protective for mood, in general, and for depression in particular [8,9]. In older people, it has been also found that the adoption of this healthy dietary pattern reduces the risk of cardiovascular diseases, as well as cancer, and the incidence of chronic diseases such as Alzheimer’s and Parkinson’s [10,11,12,13], being associated with a higher longevity [14,15]. Thus, the adherence to the MD contributes to the promotion of a positive health-related quality of life and general lifelong well-being [10,12].

However, although the MD has shown to be one of the best dietary patterns worldwide, disparities in the levels of adherence to this alimentary pattern have been found in previous research [7,16]. Several factors, in which psychosocial dimensions play a main role, seem to modulate the levels of adherence in diverse populations, but especially in adolescents [7,16]. In this regard, recent research has demonstrated that high social stressed individuals, such as bullied adolescents, exhibit a lower adherence to the MD [8]. Probably, high levels of isolation and loneliness characteristic of this population could be related with the developing of poor dietary habits, and therefore, a lower adherence to the MD. However, no previous studies have evaluated the possible association between loneliness and levels of adherence to the MD. Loneliness, as a subjective emotional state which includes dissatisfaction with the social relationships (e.g., low-quality friendships, no friendships) and feelings of disconnection with the world [17,18], emerge as a common and intense phenomenon during adolescence [19]. In recent years, adolescents’ social life has changed, mostly influenced by communication technologies [20]. In this regard, social interaction between adolescents occurs frequently through social networking sites, reducing face-to-face interaction time [20]. The growing popularity of this current way of communication could negatively affect their social behavior and interpersonal relationships [20]. Therefore, these changes in their lifestyle and daily routines could explain the dramatic increase of loneliness feelings in adolescent population [20].

Several theories have been developed to explain the relationship between loneliness and poor dietary habits. From a psychological perspective, one of the most studied explanations is based on the intake of ‘comfort foods’ as an escape coping strategy [21,22]. In the case of isolated individuals, the consumption of highly palatable and calorically-dense food could function as a coping strategy oriented to the reduction of negative psychological consequences derived from high perceived loneliness. The consumption of these type of foods has demonstrated to activate the brain reward circuitry [23], alleviating the stress perception momentarily but promoting the development of an emotional-eating behavior, a behavioral pattern classically related to poorer health outcomes [23]. The biological mechanisms that have been proposed to explain this association are based on the effects of the high cortisol secretion derived from the stress of social isolation [24]. High cortisol release has been related to high leptin concentrations, due to resistance mechanisms [24]. This fact could promote a dysregulation of the appetite and satiety biological mechanisms, and hence, reinforce the development of an emotional-eating behavior [23,24].

Alternatively, the Social Baseline Theory (SBT), based on a sociobiological perspective, propose an interactive effect between social interaction and human biological functioning [25]. This theory suggests that the human brain anticipates the development of social relationships as a protective factor to reduce risks and diminish the needed effort to reach several goals [25]. In this regard, the brain is phylogenetically constructed to get benefit from and promote social networks, as in social groups there is high protection to several environmental risks and the efforts to reach goals could be shared from several members of a group [25]. For that reason, in this theory, social relationships play a main role in several physiological processes related to the body homeostasis, such as eating behavior. In this sense, a recent research has evaluated how the lack of social relationships, characterized by high levels of loneliness, could be related with dysfunctional eating behavior [26,27].

The consequences of loneliness are diverse, having found several relationships between loneliness and poorer health outcomes involving sleep quality, depressive symptoms, and stress responses [28,29,30]. Taking into account that there is a widespread scientific evidence about the association between affective processes and eating habits, the development of negative dietary patterns could be a plausible mechanism in the association between negative affect and stress derived from loneliness with negative health outcomes [31,32,33]. In this regard, studies carried out with adolescents have shown that the daily stress is inversely linked to their eating patterns quality [21,34,35]. Thus, depressive mood, stress, and loneliness feelings experienced by adolescents could result in poorer nutritional habit choices, fundamentally the consumption of fast food and processed food [35,36,37], and therefore, a health status deterioration.

To sum up, the lifestyle changes in the last years could negatively affect the psychological health and dietary patterns in adolescents. An increase in feelings of loneliness and the stress perceived could have a negative impact on dietary habits in this population. Although previous research has analyzed the association between loneliness and eating behavior, to our knowledge, no previous research has evaluated the possible association between loneliness and the levels of adherence to a healthy eating pattern, such as the MD. Thus, the aim of this work was to identify the relationship between perceived loneliness, stress, dietary patterns, and adherence to the MD in adolescents.

## 2. Materials and Methods

### 2.1. Procedure

This study is part of a large-scale study on the MD adherence, wellbeing, and victimization carried out in schools in the Mediterranean city of Alicante (Spain). The study was approved by the Ethics Committee of the University of Alicante (UA-2015-10-13), and parents provided consent to the participation of their children prior to data collection. All subjects gave their written informed consent for inclusion before they participated in the study. The participants were 527 high school students. Students who assented to participate anonymously completed a battery of questionnaires in a paper–pencil format. The distribution and completion of questionnaires was overseen by research assistants during the second and third trimesters of the 2015/2016 academic year and the process took 60 to 70 min. Inclusion criteria for the students were: (1) being present in the classroom on the day of the survey, (2) being able to read and complete the questionnaires on their own, and (3) presenting an informed consent form signed by their parents allowing participation. Participants were only retained in the final sample if they had completed all questionnaires.

### 2.2. Measures

#### 2.2.1. Dietary Patterns and Adherence to the Mediterranean Diet

Dietary patterns and adherence to MD was evaluated with the Mediterranean Diet Quality Index for children and teenagers (KIDMED) [38,39]. KIDMED is a questionnaire originally designed in Spain, and since employed in numerous international studies, for assessing adherence to the Mediterranean diet in children and young people. Consisting of 16 questions rated on a scale ranging from 0 to 12, this tool can be self-administered or administered by an interviewer following a standard protocol. Dietary items that were included in the questionnaire evaluate the characteristic dietary pattern of the MD, a recognized healthy dietary pattern, including items referring to the frequency of the consumption of fruits and vegetables, pulses, cereals, dairy products, and fish, the use of olive oil in cooked meals at home, and questions regarding breakfast consumption and the characteristics of the breakfast usually consumed (if it includes dairy products, baked goods, or pastries). The questionnaire also included two items related to the frequency of sweets and fast food consumption. The total score on the questionnaire is classified into three levels: ≥8, indicating an ‘optimal’ Mediterranean diet; 4–7, that improvement is needed to adjust intake to Mediterranean patterns; and ≤3, very low diet quality. This questionnaire has demonstrated adequate test–retest reliability [40] and construct validity [41] in previous research. In the present study, Cronbach’s alpha for the total scale was 0.71.

#### 2.2.2. Perceived Loneliness

To assess the frequency of perceived loneliness, we asked the following ad-hoc question with corresponding four-point Likert-type response scale “Do you ever feel alone?”, with responses ranging from “I never feel alone” to “I always feel lonely”. The higher the score the higher the perceived loneliness.

#### 2.2.3. Stress Perception

Stress was evaluated with the Perceived Stress Scale [42]. This questionnaire is composed of four items scored on a five-point Likert scale, ranging from “never” to “very often”. The total score is obtained by summing across all four items (maximum = 16). Higher scores are indicative of greater stress. The Spanish adaptation developed by Herrero and Meneses [43] employed in the present study has demonstrated adequate psychometric properties. Cronbach’s alpha in this study was 0.81.

### 2.3. Data Analysis

Pearson’s correlations were employed to evaluate the relationships between loneliness, stress, dietary habits, and adherence to the MD. Participants were divided into three groups based on scores about the frequency of feelings of loneliness (“never or rarely” low perception *n* = 88, “many times” middle perception *n* = 134 and “always” high perception *n* = 305). Chi-square analyses were performed to evaluate differences between these groups in dietary habits. Hierarchical linear regression analysis was used to determine the predictive value of loneliness and stress on adherence to MD. To test the mediation effect of stress on the relationship between loneliness and adherence to MD, the macro PROCESS by Hayes was employed [44]. KIDMED and perceived loneliness scores were entered into the correlation, regression, and mediation models as continuous variables. All statistical analyses were performed using SPSS (International Business Machines Corporation (IBM), Armonk, NY, USA), Statistics for Windows, Version 24.0, considering any *p* < 0.05 as significant.

## 3. Results

### 3.1. Participant Characteristics

The participants were 527 high school students (54.5% females; 45.5% males) ranging in age from 12 to 17 years (M = 14.43, SD = 1.52). Regarding gender and age distributions in each group depending on perceived loneliness scores, the group of low perceived loneliness is composed by 52 (40.9%) females and 36 (40.9%) males, with a mean age of 14.42 (SD = 1.56); the group of middle perceived loneliness by 71 (53%) females and 63 (47%) males with a mean age of 14.35 (SD = 1.52) and the group of high perceived loneliness by 164 (53.8%) females and 141 (46.2%) males, with a mean age of 14.46 (SD = 1.50). Frequency and percentage and mean and standard deviation in dietary patterns, adherence to the MD, perceived loneliness and stress for all participants are summarized in Table 1.

### 3.2. Relationship Between Loneliness and Stress with Dietary Patterns and Adherence to the MD

Pearson’s correlations between perceived loneliness, stress, dietary habits and adherence to the MD are presented in Table 2. Both perceived loneliness and stress were negatively associated with all of the analyzed dietary patterns, except in the case of fast food, sweets, commercially baked goods, and breakfast consumption in which the associations were positive (Table 2).

### 3.3. Differences Between Adolescents Depending on the Frequency of Perceived Loneliness in Dietary Patterns and Adherence to the MD

To evaluate differences in dietary patterns and adherence to the MD depending on the frequency of perceived loneliness, adolescents were divided in three groups (Table 3). Significant differences between groups were found in all of the analyzed dietary patterns (*p* < 0.05), except in the case of breakfast and sweets consumption. In the case of adherence to the MD, differences between groups were also found (*p* < 0.01). In all cases, the group with higher feelings of loneliness showed poor dietary habits and lower adherence to the MD in comparison to those adolescents with middle or lower perception (Table 3).

### 3.4. Prediction Ability of Loneliness and Stress on Adherence to the MD

With the aim to identify the ability of perceived loneliness and stress to predict adherence to the MD, a hierarchical regression model was conducted. In order to control the possible confounding effects of age and sex, these variables were included in the first step. Perceived loneliness and stress were included in the second and third step respectively. In the first step, when age and sex were introduced, neither revealed a significant predictor. In the second step, when perceived loneliness was introduced, this factor was found to be significant. In the third step, when stress was included in the model, this variable was a significant predictor. In this final step, age, sex and perceived loneliness were not significant predictors of adherence to the MD (Table 4).

### 3.5. Mediation Effect of Stress on the Association Between Perceived Loneliness and Adherence to MD

**Figure 1:**
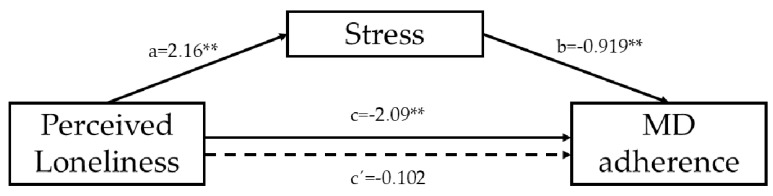
Graphical representation of the mediation effect of Stress in the association between perceived loneliness and adherence to the MD. The numerical values correspond to the unstandardized regression coefficients. Dashed line represents the mediated association. ** *p* < 0.01.

Stress was tested as a mediator of the association between perceived loneliness and adherence to the MD. Results from mediation analyses indicated that, the total effect of perceived loneliness on adherence to the MD was significant (b = −2.09, SE = 0.096, *p* = 0.00001). Perceived loneliness, in turn, predicted stress (b = 2.16, SE = 0.090, *p* = 0.00001). The mediator variable, stress, was significant predictor of adherence to the MD (b = −0.919, SE = 0.023, *p* = 0.00001). The examination of the indirect effect of perceived loneliness on adherence to the MD through the stress effect, revealed a significant mediation (indirect effect = −1.98 95% CI for bias correct lower level = −2.195, upper level = −1.785). When stress was introduced in the model as a mediator, the association between perceived loneliness and adherence to the MD did not reach statistical significance (b = −0.102, SE = 0.070, *p* = 0.1493) suggesting a full mediation effect of stress in this association. The overall model is statistically significant, F (3,523) = 156.509, *p* = 0.00001, *R*^2^ = 0.4731 (Figure 1). Age and sex were covariated in the model, but did not reveal a significant connection. These results indicate that higher levels of perceived loneliness are related to a lower adherence to MD trough the effects of higher stress levels.

## 4. Discussion

The present study examined (i) the association between loneliness, stress, dietary patterns and adherence to the MD, (ii) differences in dietary patterns and MD adherence between adolescents based on their frequency of perceived loneliness, (iii) the prediction ability of perceived loneliness and stress on adherence to the MD, and (iv) the mediation effect of stress in the relationship between perceived loneliness and adherence to the MD. The obtained results exhibit a negative association between perceived loneliness and stress with healthy dietary patterns and adherence to the MD. With regard to the specific differences between adolescents with low, middle, and high perceived loneliness, statistical analyses showed poor dietary habits and low adherence to the MD in the group of high perceived loneliness in comparison to those adolescents with medium and low perceived loneliness. Hence, regression and mediation analyses showed that loneliness predicts significant adherence to MD, through the mediation effect of stress levels.

As previously explained, the MD has been recognized over the years as one of the best dietary patterns, due to its preventive effects against many diseases. With all this, the MD contributes to the maintenance of good health and a better quality of life [10]. However, despite all the beneficial effects of the MD, there is currently a progressive loss of this eating pattern, particularly among the young adolescent population. Although the great influence of Westernized diets, fast food, and food globalization is undeniable [45]; other variables seem to be also on the basis of this phenomenon, mostly psychosocial and affective factors such as loneliness, stress, or depression [4,7,16]. These factors have been directly related to an alteration of eating habits producing a progressive loss of adherence to the MD. In this regard, this study shows for the first time that adolescents with greater feelings of loneliness, develop a poor adherence to the MD. Concretely, they showed a higher consumption of fast food, industrial pastries and sweets or candies several times a day; and lower fruit, cereal, pulses, and vegetables intake. These results coincide with those found in other studies in which adolescents with higher levels of loneliness show addictive patterns in the consumption of highly processed foods, rich in sugars, fats, and salt [26,46]. Hence, loneliness has previously been related to the development of eating disorders, such as binge eating or obesity [47]. Furthermore, these effects of loneliness on diet are not specific of Mediterranean regions, taking into account that previous research have also found a negative effect of high perceived loneliness on dietary patterns in non-Mediterranean countries [48,49,50]. Concretely, social isolation has demonstrated to be a risk factor for malnutrition and the development of inadequate alimentary habits in countries of Eastern Europe and North America, among others [48,49,50].

However, the association between loneliness and poor dietary habits is not direct, as obtained results demonstrated that is mediated by high stress levels. Although the mechanisms which underlie this association remain unclear, based on our results, the stress derived from high levels of loneliness could be on the basis of this association, modulating this relationship. It has been demonstrated that loneliness promotes high stress levels in several populations, but especially in adolescents [51,52,53]. In a like manner, a recent systematic review and meta-analysis has demonstrated a close relationship between stress and eating behaviors [54]. In this regard, this study has shown that high stress levels during childhood and adolescence promote unhealthy eating behaviors, based on the increase in the consumption of some type of food and decrease of other types. Concretely, high stress levels have been related to the intake of high-fat or palatable food, and the reduction of the consumption of fruits and vegetables, [21,22]. Several mechanisms from a psychobiological perspective have been proposed to explain these results in previous studies [21,22].

First, it has been previously demonstrated that highly stressed adolescents tend to develop an unhealthy dietary pattern, characterised by a higher intake of more sweets and fatty foods than fruit and vegetables [21,22]. Based on the theory of ‘comfort foods’, the intake of foodstuff rich in sugar and fats could serve as an escape coping strategy, due to the emotional component of eating and the rewarding effects of this type of food, reducing perceived stress [22]. This could be explained by the high content of fats, energy, and sugar of this type of foodstuff. However, attending to the poor nutritional characteristics of these type of aliments, the coping effects could be based more on psychological perception rather than on real positive effects of these type of foods, attending their nutritional composition [22]. In this regard, as has been widely demonstrated in previous research [55], specific eating behaviors and the consumption of specific types of food serve as a coping strategy, reducing stress levels and negative emotions derived from stressful situation such as the characteristic of loneliness [47,55]. This fact indicates that adolescents could counteract their feelings of loneliness by eating very palatable foods rich in sugars to give them a sense of well-being. These mechanisms would explain the significant risk factor that supposes loneliness during adolescence for the development of poor dietary habits, and hence, for the promotion of negative health outcomes.

As has been previously indicated, complementary explanations are based on sociobiological theories, such as SBT [25]. This theory, based on the effects of social interaction on physiological functioning, explains how social relationships could be protective factors for the body homeostasis maintenance [26]. In this sense, previous studies have demonstrated the harmful effects of the lack of social relationships, characterized by high levels of loneliness, on eating behavior [26]. In this regard, recent research has demonstrated how perceived loneliness was significantly associated with elevated intake of sugary beverages [26]. Following the principles of SBT, authors explained their results attending the brain metabolic needs. When the individual develops a self-perception of isolation, with a lack of social relationships, there is an increase of cognitive and psychological effort to conserve energy as there is a perception of fewer available resources from the social environment. This fact could promote the intake of high sugary beverages, in order to maintain an adequate brain metabolism by optimal glucose levels. Lonely people could have higher neuronal metabolic activity, with high glucose requirements, and hence consume more sugar in comparison to individuals with better social relationships. A recent study has corroborated these conclusions through the analysis of blood glucose levels in individuals with high social avoidance [27]. In this study, individuals with social avoidance tendencies exhibited higher basal blood circulation glucose and higher glucose consumption. Although these dysfunctional eating behaviors could correspond with an evolutionary mechanism aimed to maintain the body homeostasis and neuronal activity in an optimal range, these eating patterns could have several negative consequences for the health of adolescents. Although the mechanisms explained in this theory could be plausible explanations of the obtained results, the fact that the association between loneliness and adherence to the MD was mediated by stress levels highlights the determinant effects of this variable on dietary patterns.

Another biological mechanism that could be involved in the obtained findings is based on cortisol release. Attending that stress mediates the association between loneliness and lower adherence to the MD, high cortisol secretion from the high stress levels derived from loneliness perception could be related to dysfunctional eating habits too. Recent research indicates that leptin may play a role in the association between psychosocial stress and emotional eating characteristic of stressed individuals [24]. In this regard, chronic stress induced by high perceived loneliness promotes the secretion of high cortisol levels, and in turn, high leptin concentrations, due to resistance mechanisms [24]. This fact could be associated with greater intake of comfort food and explains, in part, the poor dietary habits of adolescents with high perceived loneliness as a possible chronically stressed population.

Although the present study entails an advance in the comprehension of the mechanisms involving the association between loneliness, stress, dietary habits, and adherence to the MD in adolescents, some limitations should be considered. Firstly, the cross-sectional design of the study does not allow us to establish causal relationships between the variables studied. Longitudinal studies are needed to explore how loneliness could predict adherence to the MD and the development of specific dietary habits in adolescents. Secondly, dietary information from participants was based on self-report which may be subject to error, in particular, underreporting. Furthermore, socioeconomic status, family structure, and Body Mass Index—variables that might influence dietary patterns in this population—were not evaluated in the present study. Future studies should replicate the obtained results controlling for these possible confounders in order to evaluate the effect of these factors on loneliness, stress, and adherence to the MD. However, no previous research has evaluated the relationship between loneliness and specific healthy dietary pattern, such as the MD. To the best of our knowledge, this study is the first in analyzing the specific association between perceived loneliness, stress, and adherence to the MD in adolescents. Hence, the obtained results in the present study improve the understanding on how psychosocial variables, such as loneliness, has an important effect on dietary habits in this population. This fact is especially relevant in order to identify specific profiles of individuals in high risk for the development of poor dietary habits, such as adolescents with high perceived loneliness.

## 5. Conclusions

Considering that from an evolutionary perspective, humans are social by nature, loneliness has severe negative consequences for the health and quality of life. As has been previously demonstrated, high stress levels derived from loneliness could be on the basis of this health deterioration. However, our results point out the possible main role of dietary patterns in these negative consequences on health derived from loneliness and stress. Attending our main findings, those adolescents who perceive high levels of loneliness with more frequency suffer from high stress levels, and develop poor dietary patterns, based on the reduction in the consumption of healthy food and the engagement in the intake of processed food with low nutritional value. Hence, this population showed lower adherence to a healthy dietary pattern such as the MD. Several hypotheses based on the consumption of processed food, high sugar intake, and palatable food as coping strategies to face with the stress derived from high perceived loneliness could be plausible explanations of the obtained results. The repetitive and abusive consumption of these foods has been demonstrated to be closely related to various health problems, such as overweight and obesity, diabetes, cardiovascular problems, systemic inflammation, hyperactivity, or a worsening of the health-related quality of life [56,57]. Bearing in mind previous research, it is evident that the MD and its characteristic dietary pattern has beneficial health effects, attending to the food groups characteristic of this dietary pattern—such as fruits, vegetables, nuts, legumes, and fish—favor an adequate intake of essential nutrients, mono-unsaturated and polyunsaturated fats, antioxidants and vitamins, thus improving health status [45,58]. Considering that isolated adolescents develop a poor adherence to the MD pattern, future studies should evaluate the relationship between perceived loneliness, dietary habits, and health status in this population, identifying the possible underlying mechanism of the lower adherence to the MD diet in the characteristic health deterioration of isolated adolescents.

## Figures and Tables

**Table 1:** Baseline characteristics of the sample in dietary patterns, adherence to the MD, perceived loneliness, and stress.

Variable/Characteristics (*n* = 527)			
**Dietary patterns**	Fruit or fruit juice daily	No	111 (21.1%)
		Yes	416 (78.9%)
	Second serving of fruit daily	No	260 (49.3%)
		Yes	267 (50.7%)
	Fresh or cooked vegetables daily	No	173 (32.8%)
		Yes	354 (67.2%)
	Fresh or cooked vegetables >1/day	No	306 (58.1%)
		Yes	221 (41.9%)
	Regular fish consumption (at least 2–3/week)	No	200 (38%)
		Yes	327 (62%)
	Fast-food (hamburger) restaurant >1 week	No	368 (69.8%)
		Yes	159 (30.2%)
	Pulse consumption >1/week	No	181 (34.3%)
		Yes	346 (65.7%)
	Pasta or rice almost daily (≥5/week)	No	216 (41%)
		Yes	311 (59%)
	Cereal or cereal product for breakfast	No	210 (39.8%)
		Yes	317 (60.2%)
	Regular nut consumption (at least 2–3/week)	No	252 (47.8%)
		Yes	275 (52.2%)
	Use of olive oil at home	No	34 (6.5%)
		Yes	493 (93.5%)
	No breakfast	No	387 (73.4%)
		Yes	140 (26.6%)
	Dairy product for breakfast	No	175 (33.2%)
		Yes	352 (66.8%)
	Commercially baked goods or pastries for breakfast	No	368 (69.8%)
		Yes	159 (30.2%)
	Two yogurts and/or 40 g cheese daily	No	313 (59.4%)
		Yes	214 (40.6%)
	Sweets and candy several times a day	No	451 (85.6%)
		Yes	76 (14.4%)
**Adherence to the MD**	Low adherence to the MD		103 (19.5%)
	Medium adherence to the MD		197 (37.4%)
	High adherence to the MD		227 (43.1%)
**Perceived Loneliness**			3.35 ± 0.89
**Stress**			8.93 ± 2.67

**Table 2:** Patterns of associations between dietary patterns, adherence to MD, perceived loneliness, and stress.

	Loneliness	Stress
Fruit or fruit juice daily	−0.343 **	−0.643 **
Second serving of fruit daily	−0.443 **	−0.561 **
Fresh or cooked vegetables daily	−0.366 **	−0.490 **
Fresh or cooked vegetables >1/day	−0.456 **	−0.552 **
Regular fish consumption (at least 2–3/week)	−0.304 **	−0.448 **
Fast-food (hamburger) restaurant >1 week	0.082	0.097 *
Pulse consumption >1/week	−0.287 **	−0.314 **
Pasta or rice almost daily (≥5/week)	−0.223 **	−0.306 **
Cereal or cereal product for breakfast	−0.333 **	−0.473 **
Regular nut consumption (at least 2–3/week)	−0.203 **	−0.282 **
Use of olive oil at home	−0.130 **	−0.324 **
No breakfast	0.027	0.109 *
Dairy product for breakfast	−0.385 **	−0.595 **
Commercially baked goods or pastries for breakfast	0.100 *	0.051
Two yogurts and/or 40 g cheese daily	−0.265 **	−0.363 **
Sweets and candy several times a day	0.085 *	−0.084 *
Adherence to the MD	−0.686 **	−0.930 **

** *p* < 0.01, * *p* < 0.05.

**Table 3:** Differences between groups on dietary patterns and adherence to the MD based on the frequency of feeling of loneliness.

		Perceived loneliness			
		Low (*n* = 88)	Middle (*n* = 134)	High (*n* = 305)	
Fruit or fruit juice daily	No	1 (0.9%)	3 (2.7%)	107 (96.4%)	χ^2^ = 85.628, *p* = 0.0001
	Yes	87 (20.9%)	131 (31.5%)	198 (47.6%)	
Second serving of fruit daily	No	11 (4.2%)	34(13.1%)	215 (82.7%)	χ^2^ = 133.167, *p* =0.0001
	Yes	77 (28.8%)	100 (37.5%)	90 (33.7%)	
Fresh or cooked vegetables daily	No	5 (2.9%)	17 (9.8%)	151 (87.3%)	χ^2^ = 92.544, *p* = 0.0001
	Yes	83 (23.4%)	117 (33.1%)	154 (43.5%)	
Fresh or cooked vegetables >1/day	No	22 (7.2%)	39 (12.7%)	245 (80.1%)	χ^2^ = 147.750, *p* =0.0001
	Yes	66 (29.9%)	95 (43%)	69 (27.1%)	
Regular fish consumption (at least 2–3/week)	No	12 (6%)	32 (16%)	156 (78%)	χ^2^ = 55.915, *p* =0.0001
	Yes	76 (23.2%)	102 (31.2%)	149 (45.6%)	
Fast-food (hamburger) restaurant >1 week	No	67 (18.2%)	101 (27.4%)	200 (54.3%)	χ^2^ = 6.238, *p* = 0.044
	Yes	21 (13.2%)	33 (20.8%)	105 (66%)	
Pulse consumption >1/week	No	9 (5%)	30 (16.6%)	142 (78.5%)	χ^2^ = 51.369, *p* = 0.0001
	Yes	79 (22.8%)	104 (30.1%)	163 (47.1%)	
Pasta or rice almost daily (≥5/week)	No	24 (11.1%)	32 (14.8%)	160 (74.1%)	χ^2^ = 39.650, *p* = 0.0001
	Yes	64 (20.6%)	102 (32.8%)	145 (46.6%)	
Cereal or cereal product for breakfast	No	10 (4.8%)	32 (15.2%)	168 (80%)	χ^2^ = 73.571, *p* = 0.0001
	Yes	78 (24.6%)	102 (32.2%)	137 (43.2%)	
Regular nut consumption (at least 2–3/week)	No	30 (11.9%)	43 (17.1%)	179 (71%)	χ^2^ = 34.375, *p* =0.0001
	Yes	58 (21.1%)	91 (33.1%)	126 (45.8%)	
Use of olive oil at home	No	2 (5.9%)	2 (5.9%)	30 (88.2%)	χ^2^ = 13.795, *p* = 0.001
	Yes	86 (17.4%)	132 (26.8%)	275 (55.8%)	
No breakfast	No	62 (16%)	97 (25.1%)	228 (58.9%)	χ^2^ = 0.748, *p* =0.688
	Yes	26 (18.6%)	37 (26.4%)	77 (55%)	
Dairy product for breakfast	No	4 2.3(%)	16 (9.1%)	155 (88.6%)	χ^2^ = 102.574, *p* = 0.0001
	Yes	84 (23.9%)	118 (33.5%)	150 (42.6%)	
Commercially baked goods or pastries for breakfast	No	68 (18.5%)	102 (27.7%)	198 (53.8%)	χ^2^ = 8.323, *p* =0.016
	Yes	20 (12.6%)	32 (20.1%)	107 (67.3%)	
Two yogurts and/or 40 g cheese daily	No	33 (10.5%)	63 (20.1%)	217 (69.3%)	χ^2^ = 43.475, *p* = 0.0001
	Yes	55 (25.7%)	71 (33.2%)	88 (41.1%)	
Sweets and candy several times a day	No	82 (18.2%)	114 (25.3%)	255 (56.5%)	χ^2^ = 5.111, *p* = 0.078
	Yes	6 (7.9%)	20 (26.3%)	50 (65.8%)	
Low adherence to the MD		0 (0%)	0 (0%)	103 (100%)	χ^2^ = 418.856, *p* =0.0001
Medium adherence to the MD		10 (5.1%)	2 (1%)	185 (93.9%)	
High adherence to the MD		78 (34.4%)	132 (58.1%)	17 (7.5%)	

**Table 4:** Parameter estimates of age, sex, perceived loneliness, and stress as predictors of adherence to the MD.

**Model 1**	**β**	***R*^2^**	**Δ*R*^2^**
Age	0.012		
Sex	0.067		
F (2,526) = 1.170, *p* = 0.311		0.004	0.004
**Model 2**	**β**	***R*^2^**	**Δ*R*^2^**
Age	0.026		
Sex	0.048		
Perceived Loneliness	−0.685 **		
F (3,526) = 156.509, *p* = 0.0001		0.470	0.469 **
**Model 3**	**β**	***R*^2^**	**Δ*R*^2^**
Age	−0.003		
Sex	0.012		
Perceived Loneliness	−0.034		
Stress	−0.905 **		
F (4,526) = 837.265, *p* = 0.0001		0.864	0.392 **

** *p* < 0.001.
